# A Comparison between Severe Suicidality and Nonsuicidal Self-Injury Behaviors in Bipolar Adolescents Referred to a Psychiatric Emergency Unit

**DOI:** 10.3390/brainsci11060790

**Published:** 2021-06-15

**Authors:** Gabriele Masi, Ilaria Lupetti, Giulia D’Acunto, Annarita Milone, Deborah Fabiani, Ursula Madonia, Stefano Berloffa, Francesca Lenzi, Maria Mucci

**Affiliations:** IRCCS Stella Maris, Scientific Institute of Child Neurology and Psychiatry, Calambrone, 56128 Pisa, Italy; ilariaL89@otmail.it (I.L.); gdacunto@fsm.unipi.it (G.D.); annarita.milone@fsm.unipi.it (A.M.); deborah.fabiani@gmail.com (D.F.); madoniau@mail.com (U.M.); stefano.berloffa@fsm.unipi.it (S.B.); francesca.lenzi@fsm.unipi.it (F.L.); maria.mucci@fsm.unipi.it (M.M.)

**Keywords:** suicidality, non-suicidal self-injuries, bipolar disorder

## Abstract

Background: Severe suicide ideation or attempts and non-suicidal self-injury (NSSI) present both differences and relevant overlaps, including frequent co-occurrence and shared risk factors. Specific categorical diagnoses, namely bipolar disorder (BD), may affect clinical features and natural histories of suicidal or not suicidal self-harm behaviour. Our study aimed to compare suicidality (severe suicidal ideation or suicidal attempts) and NSSI in referred bipolar adolescents. Methods: The sample included 95 bipolar adolescents (32 males, 63 females) aged 11 to 18 years. Thirty adolescents with suicide attempts/suicidal ideation and BD (SASIB) were compared with structured measures to 35 adolescents with NSSI and BD, without suicidal ideation or attempts (NSSIB), and to 30 adolescents with BD, without suicidal ideation or attempts or NSSI (CB). Results: Compared to CB, suicidality and NSSI were both associated with female sex, borderline personality disorder and self-reported internalizing disorders, anxiety/depression and thought disorders. The NSSI were specifically associated with somatic problems. Severe suicidal ideation and suicide attempts were associated with adverse life events, immigration, bullying, eating disorders, social problems, depressive feelings, performance and social anxiety, and feelings of rejection. Conclusions: Both shared and differential features between suicidal and not suicidal adolescents may represent possible targets for diagnostic and preventative interventions.

## 1. Introduction

Suicide is a global health problem, with at least 800,000 people dying by suicide each year; among 15–29 years old, it is the second leading cause of death [1]. Suicide attempt is defined as a nonfatal, self-directed, potentially injurious behavior with an intent to die, even if it does not result in injury, whereas suicidal ideation is thinking about, considering, or planning suicide. Nonsuicidal self-injury (NSSI) is the deliberate destruction of one’s own body without suicidal intent [2]. This category has been included in Section 3 of the Diagnostic and Statistical Manual of Mental Disorders-fifth edition (DSM-5) as a “condition for further study” [3].

The fundamental criterion for distinguishing between non-suicidal self-destructive behaviors and suicide is the intention of death [4]. Other important differences have been identified between suicide attempt and NSSI, including prevalence, frequency, lethality of methods, and attitudes towards life and death [5]. Although these differences led to the classification of NSSI and suicidal behavior as distinct clinical phenomena in the DSM-5 [3], the overlaps between self-harm with and without suicidal intent are relevant, including that many individuals engage in both behaviors [6]. This notion has led to argue that they are best conceptualised along a continuum [7], supported by growing evidence suggesting that NSSI is one of the strongest predictors of suicide attempts, above and beyond previous suicidal behavior [8]. The “continuum” model is supported by studies reporting on shared risk factors for both NSSI and suicidal behavior, including depression and borderline personality disorder [9], physical or sexual abuse [10], externalizing behaviors [9], impulsivity [11], and problems in the family [12].

Studies comparing individuals who engaged in both NSSI and suicidal behavior to individuals who engaged in NSSI, but not suicidal behavior have found that individuals who engaged in both NSSI and suicidal behavior presented more severe symptoms of psychopathology and greater psychosocial impairment than individuals who engaged only in NSSI [13,14]. Inpatient adolescents who engaged in NSSI and had attempted suicide reported greater depression, hopelessness and impulsivity [15] and greater family conflict [14] than adolescents who engaged in NSSI alone.

A study comparing youth who engaged in NSSI and suicidal behavior with those who engaged only in suicidal behavior reported that youth suicide attempters with NSSI presented more depressed symptoms, hopelessness, internalized anger, risky behaviors, loneliness than youth suicide attempters without NSSI [16]. On the contrary, other studies have found that individuals who engaged in NSSI and suicidal behavior did not report significantly greater symptoms of psychopathology than individuals who engaged in suicidal behavior but not NSSI [17,18].

Finally, few studies included comparisons between individuals who engaged in NSSI only and individuals who had made at least one suicidal attempt with unknown NSSI histories. According to these studies, individuals who had made a suicidal attempt reported more depression [19], stressful life events and more help-seeking previous the suicide attempt than individuals engaged in NSSI only [20].

Most of these studies included patients with NSSI and/or suicide attempt, irrespective of the categorical psychiatric diagnosis. It may be argued that specific psychiatric diagnoses (i.e., unipolar depression versus bipolar disorders) may strongly affect the clinical features and the natural histories of suicidal or not suicidal self-injurious behaviors. Among psychiatric disorders, bipolar disorder (BD) is characterized by the highest suicide risk [21]. Pediatric BD disrupts a child’s developmental and emotional growth, causing school failure, high-risk behaviors, substance abuse, disturbed interpersonal relationships, and hospitalizations [22]. According to the World Health Organization, BD is the fourth most burdensome illness affecting individuals between 10–24 years of age [23].

The aim of our study was to assess similarities and differences between severe suicidal ideation or suicide attempt and NSSI in a sample of bipolar adolescents, hypothesizing that different clinical and psychological features may distinguish suicidal patients from patients with NSSI.

## 2. Materials and Methods

### Sample

This was a retrospective study based on a clinical database of 95 adolescents (32 males, 63 females) aged between 11 and 18 years (mean age 14.8 ± 1.8 years), referred as inpatients to the Psychiatric Emergency Unit of our hospital between January 2018 and January 2020. Inclusion criteria were the presence of a diagnosis of BD and an IQ above 70, based on the Wechsler Intelligence Scale for Children-Fourth Edition (WISC-IV) [24]. The diagnosis of BD was based on DSM-5 criteria and a diagnostic interview, the Kiddie Schedule for Affective Disorders and Schizophrenia for School-Aged Children-Present and Lifetime Version (K-SADS-PL) [25], administered to the patient and at least one parent.

The sample was divided into three groups:

- Suicidal attempt or suicidal ideation bipolar (SASIB) group: 30 individuals (22 females, eight males, mean age 14.8 ± 1.9 years) with severe suicidal ideation (score 3 or above according to the Columbia-Suicide Severity Rating Scale (CSSRS) [26] without prior suicidal attempts, or at least one previous suicide attempt, regardless of the level of suicidal ideation;
- Nonsuicidal self-injury bipolar (NSSIB) group: 35 individuals (29 females, six males, mean age 15.0 ± 1.6 years) with NSSI, suicidal ideation absent or low (score below 3 at the CSSRS), and without prior suicide attempts. These individuals were required to meet DSM-5 criteria for NSSI disorder, in that they engaged in NSSI on at least 5 days within the past year [3];
- Control bipolar (CB) group: 30 individuals (12 females, 18 males, mean age 14.5 ± 1.9 years) without suicidal ideation and without prior suicide attempts and without NSSI.

## 3. Measures

Categorical diagnosis was assessed using the K-SADS-PL, a semi-structured interview administered by trained child psychiatrists, to diagnose BD and comorbidities, It provides a reliable and valid measurement of DSM-IV Axis I psychopathology in children and adolescents. The K-SADS-PL is administered to the parent first and then to the child. Both parties may be re-interviewed to resolve informant discrepancies. Test-retest reliability and kappa coefficients are in the good to excellent range across diagnoses [25] Clinical severity was assessed with the Clinical Global Impression Severity (CGI-S) [27] and the functional impairment with the Child Global Assessment Scale (C-GAS) [28].

CGI-S is a 7-point scale that requires the clinician to rate the severity of the patient’s illness at the time of assessment; the score ranges from 1 (normal, not at all ill) to 7 (among the most extremely ill patients).

C-GAS provides a global measure of level of functioning; the score ranges from 0 (needs constant supervision) to 100 (superior functioning). The threshold of normality is defined for scores higher than 70.

Information about the presence of familial psychiatric disorders, familial attempted or completed suicides, and familial mood disorders, was retrospectively collected using a specific questionnaire. Adverse Childhood Experiences (ACE), parental separation/divorce, bullying, family mourning and second generation immigration were assessed with an unstructured checklist.

The Columbia–Suicide Severity Rating Scale (C-SSRS) was used for the assessment of the severity of suicidal risk. This scale defines five levels of suicidal ideation:

- Level 1: Wish to be dead;
- Level 2: Non-Specific Active Suicidal Thoughts;
- Level 3: Active Suicidal Ideation with Any Methods (Not Plan) without Intent to Act;
- Level 4: Active Suicidal Ideation with Some Intent to Act, without Specific Plan;
- Level 5: Active Suicidal Ideation with Specific Plan and Intent.

According to the definition of this scale an actual suicidal attempt is a potentially self-injurious act committed with at least some wish to die, as a result of act. Intent does not have to be 100%. If there is any intent/desire to die associated with the act, then it can be considered an actual suicide attempt. There does not have to be any injury or harm, just the potential for injury or harm. Even if an individual denies intent/wish to die, it may be inferred clinically from the behavior or circumstances. Also, if someone denies intent to die, but they thought that what they did could be lethal, intent may be inferred.

Personality disorders were assessed with the Structured Clinical Interview for DSM-IV personality disorders (SCID II) [29], which includes a self-report survey of 119 items and a subsequent semi-structured interview, administered by the clinician. Each item provides a dichotomous yes/no answer (yes if the symptom is present, no if the symptom is not present). The interrater reliability and internal consistency are adequate [30].

For a dimensional assessment of psychopathology, all individuals were assessed with the Child Behavior Checklist (CBCL) [31] a 118-item scale, completed by parents, clustered in two broad-band scores, designated as Internalizing Problems and Externalizing Problems, a Total Problem Score, and with 8 different syndromes scales (Withdrawal, Somatic complaints, Anxiety/depression, Social problems, Thought problems, Attention problems, Rule-breaking behavior, Aggressive behavior). For each item, responses are recorded on a Likert scale: 0 = Not True, 1 = Somewhat or Sometimes True, 2 = Very True or Often True. The total problems scale has excellent test–retest reliability (r = 0.93) and fair internal consistency (a = 0.68), as well as excellent internal consistency, test–retest reliability, and crossinformant agreement for DSM-oriented scales [32]. We assessed also a CBCL Dysregulation Profile (CBCL-DP), based on the sum of t-scores of the three CBCL subscales, Anxiety/depression, Attention problems, and Aggressive behaviour which is an index of Emotional Dysregulation [33]. Moreover, all the patients received the Youth Self Report (YSR) [30], including 112 items, with the same possible responses and the same eight subscales of the CBCL, clustered in Externalizing or Internalizing Problems.

Patients completed a self-report measure of depressive symptomatology, the Children’s Depression Inventory (CDI) [34], including 27 items, with the following subscales: Negative Mood, Interpersonal Problems, Ineffectiveness, Anhedonia and Negative Self Esteem. Patients rate themselves based on how they feel and think in the last two weeks, with each statement being identified with a rating from 0 to 2. The CDI was translated into Italian, and normative data for the Italian CDI were collected [35].

Anxiety symptoms were assessed with the Multidimensional Anxiety Scale for Children (MASC) [36] including 39 items, with four subscales: Physical Symptoms, Harm Avoidance, Social Anxiety and Separation/Panic. Patients are asked to rate their own behavior on a 4-point scale: 0-Never true about me, 1-Rarely true about me, 2-Sometimes true about me, 3-Often true about me. Studies using the MASC have reported high retest reliability [37], favorable divergent and convergent validity [38], and good internal reliability within the four subscales [39].

The study conformed to Declaration of Helsinki; the Ethics Committee of the Hospital approved the study (Identification Code 2014/0001507).

## 4. Statistical Analyses

Descriptive analyses were used to describe demographic and clinical characteristics of the whole sample. Chi-square analyses were performed on categorical variables, and two-ways ANOVA controlling for gender on continuous variables, taking into account that there were notable gender differences between the clinical groups and the control group.

Regarding the comparisons between three groups, a post hoc Bonferroni correction was applied for the quantitative variables, while for dichotomous variables we used z test. *p* values were based on two-tailed tests with α = 0.05.

As samples size is limited, the analysis is prone to Type II Error, so Partial Eta Squared had to be calculated (and highlighted only for significative values).

To elaborate on the research question, a regression model is used to identify whether the predictors of suicidality might differ in each group: to analyze associations between suicidality, depression symptoms and biosocial values, univariate (simple) logistic regression were performed (overall percentage >70) and then a multivariate multiple logistic regression was performed in order to evidence risk factors for suicide (overall percentage >70).

## 5. Results

The three groups, SASIB, NSSIB and CB, did not differ according to mean age (14.8 ± 1.9 years, 15.0 ± 1.6 years, and 14.5 ± 1.9 years, respectively, F = 0.633, df = 2, *p =* 0.533). As regards gender ratio, there was a prevalence of females in the SASIB (F/M 22/8) and NSSIB (F/M 29/6) groups, while there was a slight prevalence of males in the CB (F/M 12/18) (χ^2^ = 14.25, df = 2, *p* = 0.001). Based on the WISC-IV scores, the three groups did not differ according to the full scale IQ, the Verbal Comprehension Index, the Perceptual Reasoning Index, the Working Memory Index and the Processing Speed Index.

About the frequency of the BD types, there was a clear prevalence of BD II than BD I in the three groups (SASIB BD I/BD II 7/23, NSSIB BD I/BD II 4/31, CB BD I/BD II 6/24), and differences among groups were not statistically significant (χ^2^ = 1.69, df = 2, *p* = 0.429).

Regarding psychiatric comorbidities, according to the K-SADS-PL and the SCID II, borderline personality disorder was significantly more frequent in the SASIB (21 [70%]) and NSSIB (23 [65.7%]) groups, compared to the CB (7 [23.3%]) (χ^2^ = 16.36, df = 2, *p* = 0.000). Attention deficit hyperactivity disorder (ADHD) was significantly more frequent in the CB group (17 [56.7%]) than in the SASIB (2 [6.7%]) and NSSIB (5 [14.3%]) (χ^2^ = 23.4, df = 2, *p =* 0.000). Eating disorders (seven [23.3%] in the SASIB group, four [11.4%] in the NSSIB and 0 [0%] in the CB) were significantly prevalent in the SASIB group than in the CB. According to the type of eating disorders, in the SASIB group there were three subjects with anorexia nervosa restrictive type, three subjects with anorexia nervosa binge/purging type and one subject with binge eating disorder; in the NSSIB group there were one subject with bulimia nervosa, one subject with anorexia nervosa restrictive type and two subjects with binge eating disorder. All the other categorical diagnoses, including Learning disabilities (χ^2^ = 3.35, df = 2, *p* = 0.188), autism spectrum disorder (χ^2^ = 3.41, df = 2, *p* = 0.182), conduct disorder (χ^2^ = 4.09, df = 2, *p* = 0.130), oppositional defiant disorder (χ^2^ = 2.5, df = 2, *p* = 0.287), anxiety disorders (χ^2^ = 1.5, df = 2, *p* = 0.472), psychotic symptoms (χ^2^ = 2.35, df = 2, *p* = 0.309), obsessive compulsive disorder (χ^2^ = 4.02, df = 2, *p* = 0.134), substance use disorder (χ^2^ = 0.38, df = 2, *p* = 0.829) and sleep disorders (χ^2^ = 2.4, df = 2, *p =* 0.301) did not differ between groups.

When clinical severity (CGI-S) and functional impairment (C-GAS) were considered, patients in the SASIB group presented greater clinical severity (CGI-S score 6.2 ± 0.5) than patients in the NSSIB (CGI-S score 4.9 ± 0.5) and CB (CGI-S score 5.2 ± 0.6) (F = 46.49, df = 2, *p* < 0.001). Patients in the SASIB group also showed greater functional impairment (C-GAS score 32.8 ± 6.1) than patients in the NSSIB (C-GAS score 44.6 ± 7.8) and CB (C-GAS score 41 ± 8.6) (F = 20.24, df = 2, *p* < 0.001) with a high significance maintained with Bonferroni correction. By correcting this analysis for gender, the statistic power is maintained (partial eta squared 0.307).

There were no statistically significant differences among groups according to the presence of familial psychiatric disorders, familial attempted or completed suicides, and familial mood disorders. Similarly, groups did not differ according to the history of adverse childhood experiences (ACE), parental separation/divorce and family mourning.

Conversely, the presence of bullying was significantly higher in the SASIB group (21 [70%]) than in the NSSIB (10 [30.3%]) (two unknown) and the CB (eight [27.6%]) (one unknown) (χ^2^ = 15.92, df = 4, *p* = 0.003). The presence of second-generation immigration (10 [33.3%] in the SASIB group, five [14.3%] in the NSSIB and two [6.7%] in the CB) was significantly higher in the SASIB group than in the CB (χ^2^ = 15.92, df = 4, *p =* 0.003). Comparing the three groups according to the total number of adverse childhood experiences, the SASIB group presented a significantly higher total number of adverse childhood experiences (4.1 ± 1.52) than the NSSIB (2.65 ± 1.37) and the CB (1.86 ± 1.27) (F = 19.8, df = 2, *p =* 0.000). For CBCL the results are listed in Table 1.

Emotional dysregulation (based on comparison in the CBCL Dysregulation Profile) did not differ among groups, and it was over-represented in all three groups, affecting 89.3% of individuals in the SASIB group, 82.9% in the NSSIB and 86.2% in the CB (Partial Eta Squared 0.019). For the YSR results are listed in Table 2.

According to the CDI the results are listed in Table 3.

Finally, regarding anxiety symptoms according to the MASC the results are listed in Table 4.

Univariate logistic regression was performed on the clinical group by taking each significative item of two ways ANOVA as dependent variable (considering an overall percentage of 70 or above). The data analysis highlighted an explanatory power for factors linked to mood (YSR internalizing, CDI total score, CDI Ineffectiveness, CDI negative self-esteem) linked to anxiety dimensions (MASC total social anxiety, anxiety dimension index), linked to global functioning assessment (CGAS) and linked to biosocial factors (total ESI number); the results are listed in Table 5.

A multiple logistic regression was conducted analysing the power of predicting suicide, finding Total ESI number, CGAS and CDI total score, taking together in the same block, account for 85% explanatory risk factor for suicide, as showed in Table 6.

## 6. Discussion

The aim of this study was to compare in a consecutive sample of bipolar adolescents referred to an emergency psychiatric unit those with suicidality but not NSSI, those with NSSIs but without suicidality, and those without the two conditions.

The three groups did not differ in age, while there was a significantly larger proportion of females in the SASIB and NSSIB groups. Females prevalence in the NSSIB group is consistent with all the available studies including both clinical and non-clinical samples [40].

Regarding the gender differences in the suicidal group, our finding is consistent with prior studies, which showed that in adolescents suicide rates are higher in males than females, while suicide attempts are more common in females [41] This phenomenon, known as the “gender paradox”, can be explained by several reasons, including the use of more lethal means in males, such as firearms and hanging methods [42], while drug poisoning is more frequent in females [43]. Another reason is the greater suicidal intentionality presented by males than females, regardless of the suicidal method [43]. Lastly, the higher rates of suicide deaths among male adolescents may be associated with a higher prevalence of externalizing disorders (with more impulsive behaviors), while females show more frequently internalizing disorders [44].

The greater presence of second-generation immigrants in the SASIB group compared to the CB is confirmed by a recent review, suggesting a higher risk of suicidal behaviour in migrant populations and ethnic minorities than in native populations [45] Risk factors among migrants and ethnic minorities are language barriers, separation from family, lack of information on the health care system, poor socio-economic status, acculturative stress, social exclusion and discrimination [45].

The significant role of bullying in the SASIB group is supported by our findings. Consistently, a longitudinal study showed that frequent exposure to bullying by peers during childhood increased the risk of deliberate self-harm at the age of 12 [46]. Moreover, this association was independent of potential confounding selection effects of maltreatment by an adult, family environmental risk factors, early behavioural and emotional problems and low IQ [46].

We found a greater prevalence of borderline personality disorder in the SASIB and NSSIB groups than in the CB. The impaired sense of identity and of interpersonal relationships, as well as the higher sensitivity to adverse life events, all core features of borderline personality disorder, can explain its association with suicidality and NSSI.

Interestingly, we also found a greater prevalence of eating disorders in the SASIB group than in the CB, as previously reported [47] In particular, suicidal behavior has been linked to bulimia nervosa and anorexia nervosa binge/purging type, where impulsivity is a central feature [48]. This finding highlights the importance of a close monitoring of specific features (sense of guilt, dysmorphophobia, perfectionism and low motivation for change) which may be more related to the development of self-injurious thoughts and behaviors.

The prevalence of ADHD in the CB group can be related to the gender differences between the groups, being males significantly more represented in CB group than SASIB and NSSI groups. Boys are indeed more than twice as likely as girls to receive a diagnosis of ADHD and a significantly higher comorbidity of ADHD in bipolar rather than non-bipolar boys is well documented [49]. Nevertheless, it is important to underline that the role of ADHD in promoting self-injurious behavior has been described particularly in depressed subjects, while suicidality in ADHD-bipolar subjects is more likely linked to bipolar disorder itself.

Comparing the three groups according to the CBCL (parent report), individuals in the CB group showed the highest levels of externalizing problems, and individuals in the NSSIB group greater externalizing problems than those in the SASIB. Rule breaking appears to be significant but lost its significance when corrected with Bonferroni post hoc analysis.

Regarding the YSR (self-report), individuals in the SASIB and NSSIB groups presented significantly higher scores on the internalizing problems, so we can hypothesize that subjective internalizing symptoms might be associated to a higher likelihood of engaging in self-harm. Neither Anxious/depressed nor Withdrawn/depressed symptoms differ between the SASIB and NSSIB groups. Anxious/depressed symptoms do differ between SASIB and CB, and NSSIB and CB with a portion of significance which is due to gender influence (significance to Gender×Group influence). In line with our results, Muehlenkamp and Gutierrez [50] found comparable levels of anxious and depressive symptoms between adolescents with NSSI and adolescents with previous suicide attempts.

The finding of greater somatic problems in the SASIB and NSSIB groups than in the CB, slightly influenced by Gender×Group effect, reflects Idenford’s [51] finding of a strong correlation between somatic compliants, NSSIB and the gateway toward suicide attempts: Idenford sustained that individuals who had complained of somatic disorders in the 12 months before an episode of self-injury were also at a higher risk of suicide; so the authors concluded that it is important to check for a possible self-injuring or suicidal risk in adolescents who access healthcare for somatic symptoms.

The finding of greater thought problems in the SASIB and NSSIB groups than in the CB underlines the importance of dysfunctional thinking (obsessive thinking, psychotic thinking, ruminative thinking) in suicidality and NSSI.

Interestingly, in the SASIB and NSSIB groups internalizing problems are reported by adolescents, but not by parents. Failure in the family to recognize these symptoms might increase the subjective suffering of adolescents and their perception of lack of familial support, leading to suicidal ideation, NSSI or suicide attempts. It should be emphasized that, in our sample, the SASIB and NSSIB groups are mainly characterized by internalizing problems, while the CB group shows a prevalence of externalizing problems. This finding might be partly explained by gender differences between groups, as we know that females are more prone to show internalizing disorders, while males present a higher prevalence of externalizing disorders.

Emotional dysrgulation (ED) is defined as a poor ability to manage emotional responses or to keep them within an acceptable range of typical emotional reactions. Interestingly, ED profiles shown by CBCL was highly represented both in CB and SASIB/NSSI groups, indicating ED as a core feature of BD itself, regardless of the association with suicidality and self-injurious behaviours [52]. This finding is widely confirmed by literature, reporting ED as a transdiagnostic trait, identified in multiple disorders such as ADHD, BD and BPD [53].

Comparing the three groups according to the CDI, the total score and all the subscales scores were significantly higher in the SASIB group than in the CB, with a noticeable gender effect weighting on the significance. In line with our results, prior work has shown that adolescents with NSSI did not significantly differ from adolescents with suicide attempt in depressive symptoms [14,50]. Furthermore, some authors [54] found significantly greater depressive symptoms in bipolar adolescents engaged in suicide attempts compared to bipolar adolescents without previous suicide attempts.

Finally, comparing the three groups according to the MASC, the total score, performance fears, total social anxiety scores and anxiety disorder index score, were significantly higher in the SASIB group compared to the CB while anxiety disorder index was also significantly higher in SASIB than in NSSIB. In line with our results, a recent study has found that comorbid anxiety increases the risk of suicidal behaviors in bipolar adolescents by 46% and, after controlling for demographic confounders and psychiatric comorbidities, the risk of association with suicidal behaviors remained statistically significant and increased by 35% [55]. In our study, anxiety disorders represent a frequent comorbidity in all three groups, but higher anxiety symptoms seem to be associated with suicidal ideation and behavior.

The analysis of results for univariate and multiple logistic regression highlights a strong predictivity for mood variables (in particular CDI total score), global functioning and adverse childhood experiences (ACE), finding that total ACE number, CGAS and CDI total score together in the same block account for 85% explanatory risk factor for suicide. These findings confirm recent assumptions that comorbid anxiety disorders and childhood maltreatment have worse outcomes of BP with increased severity and suicidal attempts [55].

Our naturalistic study presents several methodological limitations, namely the small sample size. Furthermore, the cross-sectional design of the study does not allow for firm conclusions about possible mechanisms affecting the transition from NSSI to severe suicidal ideation or attempts, as only a longitudinal, perspective design may consent an exploration of risk factors in this transition, supporting or not the continuum model between the two conditions.

In summary, in our sample of bipolar adolescents, suicidality and NSSI are both associated with female sex, borderline personality disorder and self-reported internalizing disorders, anxiety/depression and thought disorders. The NSSIs are specifically associated with somatic problems, reflecting a preferential use of the body for the manifestation of psychic distress. Severe suicidal ideation and suicide attempts are associated with adverse childhood experiences, immigration, bullying, eating disorders, lower externalizing disorders, depressive feelings, performance and social anxiety with a sense of rejection and humiliation. Lower CGAS, presence of adverse childhood experiences and higher CDI total scores, strongly predict attempting suicide. These aspects may represent possible risk factors and targets for early diagnosis, specific prevention and treatment, both in the general population and in clinical samples of bipolar adolescents, engaged or not in self-harm.

## 7. Conclusions

The clinical implications in clinical practice may be relevant. In at risk populations, such as adolescents with BD, the exploration of factors associated with suicidality, compared to those associated to NSSI, may help not only to focus intervention on specific targets, but also to prevent acute anticonservative behaviors. Indeed, at-risk adolescents often act impulsively suicidal or self-harm behaviors, without previous explicit communications, on the basis of contingent environmental factors and/or momentary internal experiences. These dynamic properties of suicidal risk account for a “fluid vulnerability” [56], with temporal fluctuation (acute dimension of risk), which may be crucial in the management and prevention of real suicidal behaviors. In these patients, environmental safety and support are fundamental in order to prevent the triggering of an acute suicidal crisis, sometimes without previous active suicidal ideation. Suicidal ideation, in fact, is not followed in the next two years by suicidal behavior in more than 92% of individuals [57]. In addition, the correlation between the anticonservative intent and the lethality of a suicidal attempt is weak in adolescents [58]. In bipolar adolescents with high suicidal risk, a specific attention to contingent and biosocial factors (namely adverse childhood experiences) implies a careful prevention of contact with potential lethal means, parental supervision of medications, avoid social isolation, and, above all, monitor possible triggers of negative emotions, such as acute anxiety, humiliation, bullying, victimization, exclusion from peers, with possible cumulative effect., with global functioning that strongly modifies individual resilience of adolescent patients.

In these at-risk patients, when the conditions explored in our study are present, learning training and exercise of coping skills for adolescents and their parents may reduce the impact of negative contingent factors, and support specific skills. Among these interventions, multimodal adapted program to adolescents of the Dialectical Behavioral Therapy (DBT) includes a multi-family group skill training setting, in the DBT-A [59] specifically aimed at reducing suicide attempts, self-injurious over time and the resort itself to hospitalization for suicidality [60]. The implementation of this clinical perspective (diagnostic and therapeutic) in mental health professionals, especially in a psychiatric Emergency Hospital Care Unit for adolescents, represents a desirable goal for improving the quality and effectiveness of the clinical practice, with the structuring and implementation of differentiated therapeutic paths and ad hoc organizational models for populations of adolescents at high suicidal risk, together with their families.

## Figures and Tables

**Table 1:** Two-way ANOVA corrected by gender: Comparison between the three groups according to the Child Behavior Checklist (CBCL) (parent reports).

	SASIB (*n* = 28) (Mean ± SD)	NSSIB (*n* = 35) (Mean ± SD)	CB (*n* = 29) (Mean ± SD)	Two Ways Anova (F;df)	*p*	Partial Eta Squared	Bonferroni-Holm (Multiple Comparisons)
Internalizing problems	71.43 *±* 7.188	71.54 *±* 6.688	69.45 *±* 6.328	(0.917;2)	ns		
Externalizing problems	64.21 *±* 8.085	69.46 *±* 8.326	70.38 *±* 8.205	Group (4.724;2)	0.049 *	0.068	SASIB/NSSIB 0.035 * SASIB/CB 0.014 *
Total problems	68.61 *±* 6.332	72.06 *±* 6.721	70.59 *±* 6.625	(2.142;2)	ns		
Anxious/depressed	75.14 *±* 11.437	70.80 *±* 10.975	68.76 *±* 8.357	(0.817;2)	ns		
Withdrawn/depressed	74.00 *±* 11.576	71.06 *±* 11.008	6.21 *±* 9.522	(1.441;2)	ns		
Somatic complaints	61.68 *±* 8.944	67.17 *±* 9.919	64.07 *±* 8.031	(2.910;2)	ns		
Social problems	64.39 *±* 6.849	65.46 *±* 8.326	64.07 *±* 7.473	(0.294;2)	ns		
Thought problems	70.79 *±* 6.839	69.71 *±* 7.775	66.66 *±* 9.401	(2.044;2)	ns		
Attention problems	63.61 *±* 9.589	68.80 *±* 12.388	66.93 *±* 10.330	(1.762;2)	ns		
Rule-breaking behavior	63.39 *±* 7.752	68.20 *±* 10.070	68.62 *±* 8.621	(3.271;2)	0.043 *	0.071	
Aggressive behavior	65.14 *±* 9.411	69.69 *±* 11.287	71.90 *±* 10.001	(0.944;2)	ns		

Legend: SASIB = Suicidal attempt or suicidal ideation bipolar; NSSIB = Nonsuicidal self-injury bipolar; CB = Controls bipolar; ns = not significant; * = *p <* 0.05.

**Table 2:** Two-way ANOVA corrected by gender: Comparison between the three groups according to the Youth Self Report (YSR).

	SASIB (*n* = 30) (Mean ± SD)	NSSIB (*n* = 33) (Mean ± SD)	CB (*n* = 26) (Mean ± SD)	Two Ways Anova (F;df)	*p*	Partial Eta Squared	Bonferroni-Holm (Multiple Comparisons)
Internalizing problems	71.37 *±* 7.188	69.33 *±* 6.688	60.38 *±* 6.328	Group (5.864;2)	0.004 **	0.124	SASIB/CB 0.001 *** NSSIB/CB 0.008 **
Externalizing problems	59.97 *±* 8.126	64.18 *±* 8.133	65.81 *±* 9.896	(3.465;2)	ns		
Total problems	67.33 *±* 9.960	68.91 *±* 7.966	64.08 *±* 8.207	(2.255;2)	ns		
Anxious/depressed	73.47 *±* 14.680	70.30 *±* 10.953	62.12 *±* 10.328	Group (5.296;2)	0.007 **	0.113	SASIB/CB 0.002 **
				Gender × Group (3.710;2)	0.029	0.082	NSSIB/CB 0.029 *
WWithdrawn/depressed	72.43 *±* 15.593	68.18 *±* 10.984	61.88 *±* 11.660	(4.683;2)	ns		
Somatic complaints	64.27 *±* 11.341	64.55 *±* 10.016	57.58 *±* 9.390	Group (4.050;2)	0.015 *	0.096	SASIB/CB 0.042 **
				Gender × Group (3.687;2)	0.029 *	0.082	NSSIB/CB 0.027 *
Social problems	65.47 *±* 11.575	63.73 *±* 10.260	58.15 *±* 8.748	(3.761;2)	ns		
Thought problems	67.33 *±* 10.880	67.39 *±* 11.233	60.35 *±* 7.440	Group (3.312;2)	0.041 *	0.074	SASIB/CB 0.036 * NSSIB/CB 0.029 *
Attention problems	64.30 *±* 9.642	68.18 *±* 10.135	64.08 *±* 9.082	(1.766;2)	ns		
Rule-breaking behavior	59.47 *±* 8.212	62.18 *±* 8.060	64.58 *±* 10.207	Gender × Group (3.884;2)	0.024 *	0.086	
Aggressive behavior	59.87 *±* 6.776	64.24 *±* 8.832	65.73 *±* 9.332	Group (3.263;2)	0.043 *	0.073	SASIB/CB 0.034 *

Legend: SASIB = Suicidal attempt or suicidal ideation bipolar; NSSIB = Nonsuicidal self-injury bipolar; CB = Controls bipolar; ns = not significant; * = *p <* 0.05; ** = *p* < 0.005; *** = *p* < 0.001.

**Table 3:** Two-way ANOVA corrected by gender: Comparison between the three groups according to the Children’s Depression Inventory (CDI).

	SASIB (*n* = 30) (Mean ± SD)	NSSIB (*n* = 17) (Mean ± SD)	CB (*n* = 28) (Mean ± SD)	Two Ways Anova (F;df)	*p*	Partial Eta Squared	Bonferroni-Holm (Multiple Comparisons)
Total	73.17 *±* 18.084	65.71 *±* 15.430	55.50 *±* 10.479	Gender (11.112;1)	<0.001 ***	0.139	SASIB/CB 0.000 ***
				Group (5.046;2)	0.009 **	0.128	
Negative mood	67.53 *±* 16.258	61.53 *±* 13.342	51.75 *±* 11.610	Gender (3.854;1)	0.054 *	0.053	SASIB/CB 0.000 ***
				Group (5.302;2)	0.007 **	0.133	
Interpersonal problems	65.13 *±* 17,290	64.06 *±* 9.915	55.79 *±* 11.262	Gender (4.610;1)	0.035 *	0.063	SASIB/CB 0.029 *
Ineffectiveness	65.87 *±* 13.718	63.12 *±* 9.980	55.07 *±* 10.760	Gender (7.161;1)	0.009 **	0.094	SASIB/CB 0.002 **
Anhedonia	63.30 *±* 13.298	56.35 *±* 13.1	52.04 *±* 8.905	Gender (5.410;1)	0.023 *	0.073	SASIB/CB 0.001 ***
				Group (3.469;2)	0.037 *	0.091	
Negative self esteem	72.63 *±* 18.011	67.35 *±* 17.755	57.54 *±* 10.868	Gender (12.650;1)	<0.001 ***	0.155	SASIB/CB 0.000 ***

Legend: SASIB = Suicidal attempt or suicidal ideation bipolar; NSSIB = Nonsuicidal self-injury bipolar; CB = Controls bipolar; ns = not significant; * = *p <* 0.05; ** = *p <* 0.005; *** = *p* < 0.001.

**Table 4:** Two-way ANOVA corrected by gender: Comparison between the three groups according to the Multidimensional Anxiety Scale for Children (MASC).

	SASIB (*n* = 27) (Mean ± SD)	NSSIB (*n* = 18) (Mean ± SD)	CB (*n* = 24) (Mean ± SD)	Two Ways Anova (F;df)	*p*	Partial Eta Squared	Bonferroni-Holm (Multiple Comparisons)
Total	60.59 *±* 13.776	52.39 *±* 13.426	49.50 ± 14.179	Gender × Group (3.449;2)	0.038 *	0.099	SASIB/CB 0.013 *
Tense/restless	61.78 *±* 12.744	59.28 *±* 13.141	55.38 *±* 12.427	(1.616;2)	ns		
Somatic/autonomic	57.33 *±* 14.085	54.61 *±* 12.733	51.79 *±* 9.991	Gender × Group (3.708;2)	0.030 *	0.105	
Total physical symptoms	60.89 *±* 12.816	57.61 *±* 13.465	53.96 *±* 10.980	Gender × Group (3.185;2)	0.048 *	0.092	
Perfectionism	42.63 *±* 10.525	40.22 *±* 12.591	39.29 *±* 10.564	(0.612;2)	ns		
Anxious coping	45.56 *±* 10.610	36.50 *±* 12.075	41.50 *±* 9.943	Gender × Group (3.818;2)	ns		
Total harm avoidance	43.48 *±* 10.067	36.61 *±* 12.103	38.71 *±* 9.603	(2.614;2)	ns		
Humiliation/rejection	63.15 *±* 13.601	54.00 *±* 12.485	52.63 *±* 12.576	(4.881;2)	ns		
Performance fears	64.70 *±* 13.044	58.56 *±* 14.076	51.00 *±* 13.062	Group (4.651;2)	0.013 *	0.129	SASIB/CB 0.001 ***
Total social anxiety	65.00 *±* 14.563	56.61 *±* 12.705	52.25 *±* 11.509	Group (3.614;2)	0.033 *	0.103	SASIB/CB 0.002 **
Separation/panic	55.89 *±* 14.750	50.78 *±* 10.664	52.08 *±* 11.938	(1.004;2)	ns		SASIB/NSSIB 0.021 * SASIB/CB 0.006 **
ADI (Anxiety disorder index)	56.96 *±* 12.936	46.33 *±* 14.471	45.67 *±* 10.320	Group (4.120;2)	0.021 *	0.116	

Legend: SASIB = Suicidal attempt or suicidal ideation bipolar; NSSIB = Nonsuicidal self-injury bipolar; CB = Controls bipolar; ns = not significant; * = *p <* 0.05; ** = *p <* 0.005; *** = *p* < 0.001.

**Table 5:** Univariate Logistic Regression.

						95% CI for Exp (B)	
	Overall%	B	Exp (B)	df	Sig	Lower	Upper
CBCL externalizing	ns						
CBCL rule breaking	ns						
YSR Internalizing	70.8	0.044	1.045	1	0.030 *	1.004	1.087
YSR anxious depressed	ns						
YSR somatic complaints	ns						
YSR Thought problems	ns						
YSR Rule breaking	ns						
YSR Aggression	ns						
CDI total	73.3	0.055	1.056	1	0.001 ***	1.022	1.091
CDI negative mood	ns						
CDI Interpersonal problems	ns						
CDI ineffectiveness	70.7	0.052	1.053	1	0.012 *	1.011	1.097
CDI Anhedonia	ns						
CDI negative self esteem	70.7	0.044	1.045	1	0.006 *	1.013	1.078
MASC Total	ns						
MASC performance fears	ns						
MASC total social anxiety	72.5	0.062	1.064	1	0.003 **	1.022	1.108
MASC Anxiety Disorder Index	71	0.071	1.074	1	0.002 **	1.026	1.123
CGAS	70.5	−0.187	0.829	1	0.000 ***	0.763	0.901
Total number of Adverse Childhood Events	78.5	0.898	2.454	1	0.000 ***	1.623	3.712

Legend: CBCL: Child Behavior Checklist; YSR: Youth Self Report; CDI: Children Depression Inventory; MASC: Multidimensional Anxiety Scale for Children; CGAS: Children Global Assessment Scale; ns = not significant; * = *p <* 0.05; ** = *p <* 0.005; *** = *p* < 0.001.

**Table 6:** Multiple Logistic Regression.

	Overall %	B	df	Sig.	Exp(B)	95% CI per EXP(B)	
						Lower	Upper
ACE_TOT	85.1	1.156	1	0.001 **	3.178	1.637	6.167
CGAS		−0.237	1	0.000 **	0.789	0.691	0.901
CDI_tot		0.052	1	0.040 *	1.054	1.002	1.108
constant		1.047	1	0.683	2.850		

Legend: ACE_TOT: Total number of Adverse Childhood Events; CGAS: Children Global Assessment Scale; CDI: Children Depression Inventory; ns = not significant; * = *p <* 0.05; ** = *p <* 0.001.

## Data Availability

The data presented in this study are available on request from the corresponding author. The data are not publicly available due to privacy.
